# Prevalence and determinants of prehospital delay among stroke patients in mainland China: A systematic review and meta-analysis of the study protocol

**DOI:** 10.1371/journal.pone.0312551

**Published:** 2024-10-24

**Authors:** Hui Deng, Xiangming Wang, Li Yin, Xianzhi Li, Yuehui Zhang

**Affiliations:** 1 North Sichuan Medical University, Nanchong, China; 2 Department of Neurology, Panzhihua Central Hospital, Panzhihua, China; 3 Meteorological Medical ResearchCentre, Panzhihua Central Hospital, Panzhihua, China; 4 Clinical Medical Research Center, Panzhihua Central Hospital, Panzhihua, China; 5 Dali University, Dali, China; San Giuseppe Hospital, ITALY

## Abstract

**Background:**

Prehospital delay is one of the most serious problems in the treatment of stroke patients. In China, although hospitals at all levels have promoted the construction of stroke centers, pre-hospital delays are still very common. As the primary cause of death and disability, stroke not only brings great harm to patients themselves, but also brings a heavy burden on social progress and economic development, it is important to understand the prevalence and determinants of prehospital delay among stroke patients. Therefore, this review aims to determine the pooled prevalence and determinants of prehospital delay in mainland China.

**Methods:**

A systematic review of eligible articles will be conducted using preferred reporting items for systematic reviews and meta-analysis (PRISMA) guidelines. A comprehensive literature search will be conducted in PubMed, Embase, Cochrane, web of science, China National Knowledge Infrastructure (CNKI), Wanfang, Weipu (VIP) and Chinese Biomedicine Iiterature databas (CBM) databases. The quality of the articles included in the review will be evaluated using the Newcastle-Ottawa Scale (NOS). The pooled prevalence of prehospital delay, and odds ratio and their 95% confidence intervals for relevant influencing factors, will be calculated using RevMan 5.3 software. The existence of heterogeneity among studies will be assessed by computing p-values of Higgins’s I^2^ test statistics and Cochran’s Q-statistics. Sensitivity analysis and subgroup analysis will be conducted based on study quality to investigate the possible sources of heterogeneity. Publication bias will be evaluated by funnel chart and by Egger’s regression test. This review protocol has been registered PROSPERO (CRD42023484580).

**Discussion:**

By collecting and summarizing information on prehospital delay among stroke patients can be a step towards a better understanding of the prevalence of prehospital delay among stroke patients in mainland China and how the associated factors influence the prevalence of prehospital delay. Therefore, a rapid, accurate diagnosis Stroke, timely pre-hospital first aid, the treatment process forward, for the patient It has great significance. This summarized finding at the national level will provide new clues for intervention to reduce the rate of pre-hospital delay of stroke patients, and is expected to further improve the treatment effect of stroke patients.

## 1. Introduction

Stroke, one of the main causes of death in mainland China [1], can be divided into ischemic and hemorrhagic stroke. Among them, ischemic stroke accounts for about 80% of all stroke patients. Timely and rapid promotion of cerebrovascular blood supply is essential for maintaining and restoring nervous system function after acute ischemic stroke (AIS). Patients with acute ischemic stroke (AIS) usually have the opportunity to receive intravenous alteplase (IVT) in the time window of 3–4.5h after symptom onset or endovascular thrombectomy (EVT) in the time window of 6h [1]. In mainland China, the percentage of AIS patients receiving IVT is relatively low, about 1 to 10 percent [1]. Hypertension is one of the main causes of hemorrhagic stroke. In clinical treatment of acute cerebral hemorrhage, the earlier the treatment, the better the prognosis. Delay in treatment will lead to further aggravated bleeding, and even respiratory failure, circulatory failure and death [2]. However, the rate of prompt treatment for ischemic stroke and hemorrhagic stroke is relatively low because most patients arrive too late, it lost the sensitive treatment in the time window. Therefore, prehospital delays place a significant medical and financial burden on patients and families.

At present, several studies have estimated prevalence of prehospital delays among stroke in main China. There are several studies that have reported relevant data on prehospital delays in some regional studies in mainland China [3–11]. Most studies were from single hospital, for example, Fang et al. [10] reported the time interval between stroke onset and hospital arrival in acute ischemic stroke patients in Shanghai, China. Yuan et al. [9] reported the analysis of time to the hospital and ambulance use following a stroke community education intervention in China. Zhu et al. [3] reported the factors associated with prehospital delay and intravenous thrombolysis in China. Or two different hospitals, for example, Kakame et al. reported the prevalence and factors associated with prehospital delay among acute stroke patients [6, 7]. These studies were limited to one or two hospitals and poorly representative. Three studies reported the prehospital delay among stroke patients in multi-city, but the three studies had small sample sizes and low credibility [3, 7, 8]. Yuan et al. [11] reported the age and geographic disparities in acute ischemic stroke prehospital delays in China at the national representative level. However, only ischemic stroke is focused and hemorrhagic stroke is ignored in this national study. The disease burden of hemorrhagic stroke is also very high in China, so the attention to the prehospital delay of hemorrhagic stroke is also significant. Therefore, it is necessary to conduct a meta-analysis evaluation prevalence of prehospital delays among stroke patients in mainland China.

Identifying and intervening the influencing factors of prehospital delay among stroke patients is very important, which can markedly reduce the occurrence rate of prehospital delay, better enhance the treatment effect and improve the prognosis of stroke patients. At present, some studies have found that there are several factors affecting the prehospital delay [3, 12–14]. Major determinants that lead to prehospital delay include individual factors (such as live alone, lack of knowledge of stroke symptoms, lower levels of education), family factors (such as transport mode, distance from hospital) and social factors (such as emergency medical services, lack extensive public education). However, studies that have focused on the factors affecting prehospital delay for stroke patients have been inconsistent. However, all the results showed that the rapid identification of cerebral infarction symptoms early on was the key to shorten the prehospital delay time. Therefore, it is necessary to conduct a meta-analysis to identify the determinants affecting prehospital delay among stroke patients in mainland China.

Therefore, this systematic review and meta-analysis will provide the estimation of the pooled prevalence and determinants of prehospital delay among stroke patients in mainland China. Provide a scientific basis for solving pre-hospital delays. Purpose of reducing prehospital delay in patients with acute cerebral infarction. At the same time, which can help the policymakers and public health professionals for decision making.

## 2. Materials and methods

### 2.1. Registration and results reporting

This protocol for this systematic review and meta-analysis has been prepared according to the Preferred Reporting Items for Systematic review and Meta-analyses (PRISMA-P) checklist [15] and this review has been registered at International Prospective Register of Systematic Reviews (CRD42023484580).

### 2.2. Study design and search strategy

**Fig 1:**
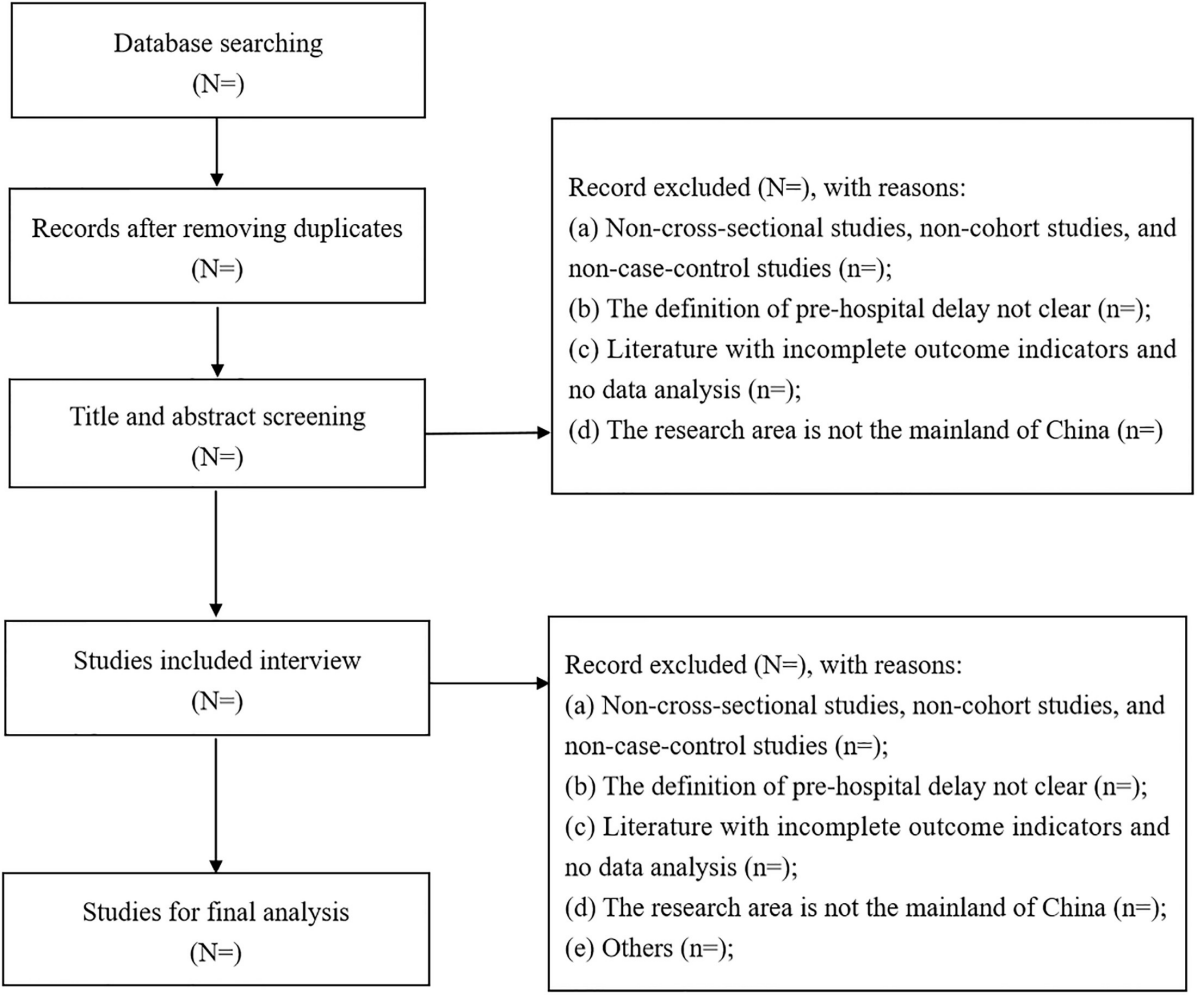
Flow chart of literature screening process.

The study is a systematic review and meta analysis which aims to identify the pooled prevalence and its determinants of prehospital delay among stroke patients in mainland China. Prior to the review process, we searched all databases to inspect if the identical systematic review and meta-analysis existed and to avoid duplication. In this review, the studies published between January 1, 2000 and December 31, 2023 in PubMed, Embase, Cochrane library, web of science, China National Knowledge Infrastructure (CNKI), Wanfang, Weipu (VIP) and Chinese Biomedicine Iiterature databas (CBM) databases will be systematically searched. Language will be limited to English and Chinese. The articles will be searched by using the following keywords: “stroke” and “prehospital delay” The searching strategy to be used for PubMed online database is given in (Table 1). We also performed a manual retrieval for reference, lists of the included articles. Grey literature will be retrieved using Google and Google Scholar. All the literature screening and selection of research procedures will be described through the use of the PRISMA flowchart (Fig 1).

**Table 1:** Draft of search strategy to be used using PubMed electronic database.

Components	Search items	Results
1	"stroke"[MeSH Terms]OR”Vascular Accident, Brain”[Title/Abstract]OR”Brain Vascular Accident’[Title/Abstract]OR”Brain Vascular Accidents”[Title/Abstract]OR “CVAs (Cerebrovascular Accident)”[Title/Abstract]OR “CVA (Cerebrovascular Accident)”[Title/Abstract]OR “Vascular Accidents, Brain”[Title/Abstract]OR “Cerebrovascular Accidents, Acute”[Title/Abstract]OR “Cerebrovascular Accident, Acute”[Title/Abstract]OR “Acute Cerebrovascular Accident”[Title/Abstract]OR “Acute Cerebrovascular Accidents”[Title/Abstract]OR “Cerebral Strokes”[Title/Abstract]OR”Cerebral Stroke”[Title/Abstract]OR” Apoplexy, Cerebrovascular”[Title/Abstract]OR” Cerebrovascular Apoplexy”[Title/Abstract]OR”Cerebrovascular Stroke”[Title/Abstract]OR” Stroke, Cerebrovascular”[Title/Abstract]OR” Cerebrovascular Accident”[Title/Abstract]OR” Cerebrovascular Accidents”[Title/Abstract]OR”Cerebrovascular Strokes”[Title/Abstract]OR”Stroke, Cerebral”[Title/Abstract]OR”Strokes, Cerebrovascular”[Title/Abstract]OR” Strokes, Cerebral”[Title/Abstract]OR” Acute Strokes;’[Title/Abstract]OR “Strokes, Acute”[Title/Abstract]OR “Acute Stroke”[Title/Abstract]OR“Stroke, Acute”[Title/AbstractOR” Strokes”[Title/Abstract]OR “Apoplexy”[Title/Abstract]	29,492
2	“pre-hospital emergency” OR “pre-hospital first aid”[Title/Abstract] OR “pre-hospital emergency care”[Title/Abstract]	632
3	“pre-hospital delay”	248
4	"determinants "AND"Prevalence"	18,709
5	“determinants”OR “influence factor”[Title/Abstract]OR “factor”[Title/Abstract]	13,476,014
	#1AND#4	192
	#3AND#5	152
	#1AND#3	13
	#1AND#3AND#5	8

### 2.3. Eligibility criteria

#### 2.3.1. Inclusion criteria

1. Population: Stroke patients.
2. Exposure: Determinants of prehospital delay.
3. Comparator: Prevalence of prehospital delays in rural and urban areas, prevalence of prehospital delays among different age group sex. subtype of stroke (ischemic stroke, hemorrhagic stroke).
4. Research type: All cross-sectional studies, cohort studies, and case-control studies focused on the prevalence and determinants of prehospital delay among stroke patients.
5. Region of studies: Mainland China.
6. Outcome: Prevalence and its determinants of prehospital delay.
7. All articles published in English and Chinese language.

#### 2.3.2. Exclusion criteria

Studies will be excluded if they include:

1. Non-cross-sectional studies, non-cohort studies, and non-case-control studies.
2. Duplicate publication.
3. The definition of pre-hospital delay not clear.
4. Literature with incomplete outcome indicators and no data analysis.
5. The research area is not the mainland of China.

### 2.4. PECO search guide

1. Population: stroke patients.
2. Exposure: Determinants of prehospital delay. The determining factor was the exposure that increased the prevalence of prehospital delays of stroke. These risk factors include the ambulance transport, emergency medical services (EMS), extensive public education, live alone, knowledge of stroke symptoms, education level, distance from hospital and so on.
3. Comparator: The reference group of each determinant in every study. Prevalence of prehospital delays in rural and urban areas, prevalence of prehospital delays among different age group, sex, subtype of stroke (ischemic stroke, hemorrhagic stroke).
4. Outcome: Prevalence and its associated determinants of prehospital delay among stroke patients in mainland China.

### 2.5. Outcome measurement

According to previous studies, prehospital delay refers to a delay of less than 3 hours from the onset of illness to arrival at the hospital [4, 9].

### 2.6. Screening and selection procedure

The entire primary screening process will be carried out through different screen criteria. At first stage, all the relevant articles will be collected and the duplicates will be identified and removed. In the second stage, the publications containing the search criteria in the title, in the keywords and in the abstract will be included. The full-text articles will be assessed following the above certain exclusion and inclusion criteria in the third phase. Throughout the process, if the full text is unavailable, the reviewers will contact the author for information about the entire article. This study includes all selected articles that are approved by both reviewers. If any disagreement appear between the two reviewers, it will be solved through negotiation, otherwise a third reviewer will be consulted if necessary.

### 2.7. Data extraction

The reviewer will independently screen the articles based on title and abstract. Then two reviewers independently extract relevant data from selected articles. If there are any differences of opinion, they will be discussed and resolved. A Microsoft spreadsheet in a predefined data extraction format will be used to gather information on author names, year of publication, study area, sample size, age range of participants, sex of participants, definitions of prehospital delay, subtype of stroke, number of patients with prehospital delay and determinants of prehospital delay among stroke patients will also be extracted.

### 2.8. Bias risk and quality assessment

Reviewers will assess the quality of the study by using the Newcastle-Ottawa Quality Assessment Scale (NOS) for cross-sectional studies, case-control studies and cohort studies [16]. The parameters used for quality evaluation are representativeness of sample (sample strategy), age, sample size, treatment time, and so on. Ultimately, each study will be scored based on 10 criteria to determine its quality. The scale will be used for evaluation the inner and outer validity, bias risk, and methodological quality of every included cross-sectional, cohort, and case-control study. The quality evaluation tool consists of three parts. The first part will focus on evaluating the methodological quality of each study, including objectives, sample size, and sampling technique. And this part will be rated on a five stars scale. In second part, studies will be rated out of two stars based on their comparability. In the third section, outcome measures and data analysis will be graded out of three stars. Studies with scores greater than or equal to 6 points will be included in the systematic review and meta-analysis of prevalence. Quality evaluation will be examined independently by four readers, and any divergences will be solved through negotiation. If differences persist, the average reviewers score will be brought into account. Likewise, in the context of influencing factors, each influencing factors with the outcome variables will be critically evaluated. Likewise cutoff points will be applied to all prevalence studies of prehospital delay among stroke. The level of risk of bias in each of the parameters will be presented separately for each study in tables in the final draft of the review.

### 2.9. Data analysis and assessment of publication bias

This study will be carried out using RevMan 5.3 and Stata Statistical Software 17. The extracted data from every study will be imported into the RevMan 5.3 software using a Microsoft Excel spreadsheet. The prevalence of prehospital delays will be determined by dividing the number of positive responses by the total number of study participants. Based on the binomial distribution formula, the standard error of prevalence will be calculated for each original article. Random effects model or fixed effects model will be used to estimate the pooled effect. Separate groups of meta-analysis will be conducted on each associated factor to examine the impact on the outcome variable.

We will assess heterogeneity among studies using p-values of Higgins’s I^2^ statistics and Cochran’s Q-statistics based on chi-square with a 5% level of significance [17] I^2^ will be calculated to assess the magnitude of the heterogeneity between studies. We will classify the heterogeneity of the studies as low, moderate, and high based on I^2^ values less than 25%, 50%, and 75%, respectively [18]. If the I^2^ greater than 50%, a random-effect model will be used for meta-analysis, otherwise a fixed-effect model will be used [17].

We will conduct sensitivity analysis and subgroup analysis based on the quality of the study to, investigate the possible sources of heterogeneity. Sensitivity analysis will assess the stability of the findings by excluding certain studies individually. The subgroup analysis will be conducted by residence, age, sex, definitions of prehospital delay, and the subtype of stroke. The funnel plots and Egger’s regression test will be employed to assess the existence of publication bias [19, 20]. The funnel plot illustrates the effect size against sample size of the studies included in the meta-analysis. A nonparametric trim and fill analysis will be used to manage the observed publication bias in estimating the number of missing studies, as well as reducing and adjusting publication bias in meta analysis [19].

## 3. Discussion

The systematic review and meta-analysis in this programme will comprehensively explore the usable proof on the prevalence of prehospital delays in stroke patients and in the meantime to identify the hazardous factors or determinants related to prehospital delay. By gathering and summarizing all the information, we can get a much more accurate picture of the prevalence of prehospital delay of in mainland China and how do correlation element affect the prevalence of this prevalence. This meta-analysis will provide scientific basis and research direction for further medical research and medical personnel.

This program can determine the current prevalence of prehospital delay stroke in China (from urban and rural areas, gender, age, and stroke subtype delays), subgroup analysis can also identify vulnerable populations for delay, thus indicating our intervention priorities. On the other hand, it suggests that the public and relevant departments should pay attention to the current problem of prehospital delay, and construct effective preventive measures, so as to contribute to reducing the prehospital delay. Secondly, by determining the determinants of the prehospital delay of stroke, it can be jointly solved from various aspects to reduce the time of hospital arrival and maximize the functional recovery after stroke, to provide the basis for the development of prehospital first aid for stroke patients. By identifying the determinants of prehospital delay in stroke and guiding the development of relevant and targeted interventions in the future, it is expected that the rate of prehospital delay will be reduced, thereby improving patient outcomes and reducing the burden of disease and socio-economic burden. This will provide important guidance for further research and healthcare practitioners.

There may be some limitations in this study. First, our assessment will only include studies published in English and Chinese. Because of the barrier of language, relevant studies in some other languages may be missed. In addition, based on search strategies and inclusion and exclusion criteria, fewer foreign studies were included in the final analysis, and the results were underrepresented in mainland China. Finally, the literature comes from different regions and hospitals. There are some differences in the emergency measures of prehospital stroke in medical centers, and the consistency of emergency measures cannot be fully guaranteed.

## Supporting information

S1 Checklist PRISMA-P (Preferred Reporting Items for Systematic review and Meta-Analysis Protocols) 2015 checklist: Recommended items to address in a systematic review protocol*. (DOC)
